# Mitigating Effect of Taurine Combined with Corona Dormancy on Oxidative Stress in *Trachinotus ovatus* Under Low-Temperature Stress

**DOI:** 10.3390/ijms26072927

**Published:** 2025-03-24

**Authors:** Siwei Liu, Kaicui Zhong, Jiamei Zhong, Xiuping Fan, Xiaoming Qin

**Affiliations:** College of Food Science and Technology, Guangdong Ocean University, National Research and Development Branch Center for Shellfish Processing (Zhanjiang), Guangdong Provincial Key Laboratory of Aquatic Products Processing and Safety, Guangdong Provincial Engineering Technology Research Center of Seafood, Guangdong Province Engineering Laboratory for Marine Biological Products, Guangdong Provincial Engineering Technology Research Center of Prefabricated Seafood Processing and Quality Control, Zhanjiang 524088, China; 13393844889@163.com (S.L.); koichui163@163.com (K.Z.); 17322913974@163.com (J.Z.)

**Keywords:** *Trachinotus ovatus*, live transportation, taurine, corona dormancy, oxidative stress

## Abstract

The purpose of the present work was to establish the transportation conditions for keeping *Trachinotus ovatus* alive by means of corona-induced dormancy in combination with taurine. It also investigated the synergistic regulatory effects on oxidative stress mitigation and immune function during low-temperature conditions and clarified the underlying mechanism. The dormancy pretreatment induced by pulsed direct current could reduce the accumulation of reactive oxygen species in fish under hypothermal and water-restricted conditions and significantly enhance the environmental adaptability of *Trachinotus ovatus*. The survival period and survival rate of *Trachinotus ovatus* were significantly increased when combined with taurine at a concentration of 70 mg/L, and the activities of enzymes related to oxidative stress also increased significantly, including catalase, superoxide dismutase (SOD), glutathione S-transferase, and so on. The underlying mechanism involved the upregulation of mRNA expression in the Nrf2/Keap1 pathway components. Furthermore, taurine supplementation was found to bolster the immune function of *Trachinotus ovatus*. Histological examinations revealed that taurine exerted protective effects on the ultrastructural integrity of the liver and gills, which were susceptible to stress-induced damage during transportation. Altogether, the concerted action of corona dormancy and taurine significantly mitigated the stress responses and tissue injuries of fish during low-temperature live transportation, thereby providing a mechanistic basis for the enhancement of live fish transportation techniques.

## 1. Introduction

Aquatic products serve as a crucial source of high-quality animal protein in daily life. Among them, as is well known, fresh fish not only has rich nutritional value and a delectable taste, but it also presents multiple unique processing approaches [1]. Currently, the sale of live fish is a value-added process, as it can generate higher profits and lower processing costs [2,3]. In particular, for the rare and precious species, their selling prices can be up to five to ten times that of other chilled or frozen aquatic products [4]. At present, fresh fish are generally kept alive by avoiding their death or minimizing mortality. Therefore, it is essential to either maintain the natural environment on which they rely for survival or adopt a series of measures to reduce their metabolic activities. During the live transportation of marine fish, they are subject to the interactive influence of multiple stress factors. For example, being startled during fishing operations, the vibrations and noises generated by machinery during live transportation, the deterioration of water quality (total ammonia nitrogen, carbon dioxide, pH value, etc.) during the in vivo preservation process, and fluctuations in environmental temperature all can induce intense stress responses in fish [5]. The above-mentioned factors lead to disorders in behavior, physiology, and metabolism, further reducing the stress resistance of fish bodies and adversely affecting their health and quality [6].

Most fish are poikilotherms, whereby their metabolic rate will be significantly reduced as the temperature decreases. Consequently, a low-temperature environment exerts a beneficial impact on prolonging the survival time of these aquatic animals. Furthermore, low-temperature water can reduce the activity level of the fish’s nervous system and diminish the energy consumption and physiological effects caused by stress responses [7]. As a result, this approach is frequently utilized for the live transportation of fish in situations with little or no water [8]. Nevertheless, prolonged exposure to low-temperature conditions often subjects fish to a series of stress responses caused by low temperatures. For instance, the rate of enzymatic reactions declines, resulting in a decreased demand for ATP and an accumulation of electrons at certain points on the respiratory chain [7]. Simultaneously, reactive oxygen species generated in response to environmental low-temperature stress disrupt cytoplasmic calcium homeostasis, leading to DNA damage, protein oxidation, lipid peroxidation, and apoptosis [9].

In order to solve the problems mentioned above, chemical and physical methods are adopted to anesthetize or put fish into a dormant state during live transportation. They not only can improve the survival rate of fish, but also reduce stress responses and maintain excellent nutritional quality. However, the chemical anesthesia method is efficient but entails the risk of drug residues, and there are certain withdrawal period limitations for fish in live transportation [10]. In physical dormancy, although the low-temperature-induced dormancy method is widely used, it requires gradient cooling acclimation before intervention, which is time-consuming. It is noteworthy that corona-induced dormancy is a green, safe, novel, and highly efficient physical dormancy mode with broad application prospects. Chakraborty et al. (2022) discovered that corona-induced dormancy technology was economically convenient and did not leave any drug residues after application, so it was suitable for replacing chemical anesthetics in aquaculture [11]. Faust et al. (2017) successfully made use of corona-induced dormancy in fish surgical operations, including breeding and cultivation, vaccine injection, and tag deployment [12]. Bouwsema et al. (2022) concluded that electric shock could offer immediate and long-lasting coma for juvenile salmon, making it appropriate for humane slaughter or subsequent live transportation and processing [13]. Nevertheless, the effect of corona-induced dormancy is of short duration, resulting in the fish being able to fully regain their vitality after 24 h. For long-term live transportation, it still cannot mitigate the damage to fish bodies caused by environmental stress [14]. Hence, on the condition of ensuring the necessary nutritional factors for animals, it is of particular significance to figure out how to apply green, safe, and non-toxic preparations or additives to address the stress issues of animals during transportation.

Taurine (2-aminoethanesulfonic acid), a sulfur-containing amino acid, predominantly exists in a free state within the interstitial fluid and intracellular fluid, and it exerts its function as a regulator by providing a certain osmotic pressure to cells [15,16]. The content of taurine in marine organisms is higher than that in mammals, and it is mainly present in tissues such as the eyes, brain, liver, muscle, and heart [17]. As a functional amino acid, taurine exerts a crucial role in regulating energy metabolism in skeletal muscle, adipose tissue, and liver tissue [9]. At the same time, taurine can serve as a neuroprotective agent and nutrient that is capable of regulating the neural transmission process, facilitating the operation and development of the brain. In addition, it has a positive impact on improving the growth condition of fish [18]. Presently, a growing number of studies have verified that taurine exerts significant functions in aspects like antioxidation, osmotic pressure regulation, and neural and immune modulation [19]. Shi et al. (2022) discovered that taurine was capable of preventing liver damage in *Monopterus albus* induced by H_2_O_2_ [20]. Its protective effect was realized through inhibiting oxidative stress, inflammation, apoptosis, and autophagy, and the optimal addition amount of taurine was 0.5%. Shi et al. (2021)’s studies demonstrated that taurine supplemented in the feed alleviated the liver oxidative damage induced by fish oil oxidation through the Nrf2-Keap1 signaling pathway based on transcriptional expression and substantially enhanced the activity of antioxidant enzymes [21]. Furthermore, exogenous taurine alleviated the inflammatory response in the intestine through the NF-κB signaling pathway based on transcriptional expression. In spite of the fact that numerous studies on the biological functions of taurine have been carried out, there remains limited knowledge concerning the response function of taurine to environmental stress in fish.

*Trachinotus ovatus*, also known as the golden pomfret, belongs to Trachinotus. It is the marine cultured fish species with the highest level of modernization and intensification in many coastal countries around the world and holds tremendous development potential [22,23]. *Trachinotus ovatus* is devoid of intramuscular spines, and its meat is tender and delicious, being rich in protein, linoleic acid, DHA, and other multiple unsaturated fatty acids. To a certain degree, it can prevent and treat various cardiovascular and cerebrovascular diseases [24,25]. *Trachinotus ovatus* is a warm-loving omnivorous migratory fish species that has a high oxygen consumption and a vigorous metabolic rate. Current studies have demonstrated that 15 °C is the most favorable survival temperature for *Trachinotus ovatus* during the process of live transportation. Fishing induces intense stress in a low-temperature environment (<13 °C), which then leads to death [14]. This is the major cause of the difficulties in its live transportation and the scarcity of live individuals in seafood markets. In recent years, the studies conducted by various scholars on *Trachinotus ovatus* have been primarily centered on freezing and preservation, with a paucity of investigations on in vivo preservation [26].

Current pretreatment protocols for marine fish transportation predominantly rely on chemical anesthesia and low-temperature-induced dormancy. Current stress-mitigation strategies predominantly rely on dietary supplementation of immunostimulants or pharmaceutical agents. However, their efficacy is nullified by mandatory pre-transport fasting protocols during live fish transportation and holding procedures. Furthermore, the drugs themselves can cause certain damage to fish bodies, since drug residues may persist. Although corona dormancy technology is more environmentally friendly, safe, and efficient, its application exhibits significant species-specific variations. Over the past decade, studies on corona dormancy have predominantly focused on freshwater species, while research on marine aquaculture organisms remains insufficient. Furthermore, the synergistic enhancement of live fish transportation through the combined use of physical corona technology and anti-stress agents has not been documented in the existing literature [20,27].

This study investigated the low-temperature live transportation of *Trachinotus ovatus* using pulsed direct current corona-induced dormancy technology combined with a taurine-supplemented protective solution. Through comprehensive analysis of physiological–biochemical parameters, oxidative stress indicators, histopathological sections, and expression profiles of the genes associated with the Nrf2-Keap1 signaling pathway, we aimed to optimize transportation parameters and elucidate the antioxidant defense mechanisms in fish under low-temperature stress. This provides a solid foundation and theoretical basis for subsequent research on the regulatory mechanisms of the antioxidant stress response in *Trachinotus ovatus* under low-temperature stress conditions.

## 2. Results

### 2.1. Influence of Synergistic Anti-Stress Agents on Live Transportation

As delineated in Table 1, comparative analysis revealed that Groups 2–17 exhibited significantly prolonged survival duration and enhanced survival rates relative to Group 1, demonstrating the efficacy of electrically induced dormancy in optimizing live transport outcomes. Furthermore, Groups 3–5 demonstrated superior survival parameters compared with Group 2, albeit with concomitant reduction in aquatic pH levels, indicating a synergistic interaction between vitamin C (VC) supplementation and corona dormancy in promoting *Trachinotus ovatus* survival during transport. Notably, the acidic transport medium (pH < 6.5) was associated with epidermal corrosion and increased susceptibility to pathogenic infections, ultimately compromising post-transport product quality and market value. Comparative analysis revealed that Groups 6–7 demonstrated significantly enhanced survival duration and survival rates relative to Group 2 (*p* < 0.05), indicating a concentration-dependent synergistic interaction between moderate–low levels of astragalus polysaccharide (APS) and corona dormancy in promoting *Trachinotus ovatus* survival during live transport. However, Group 8 exhibited reduced survival parameters, with a particularly notable decline in 48 h survival rate, attributable to the physicochemical properties of APS. The fine, yellowish-brown powder formulation at elevated concentrations induced water turbidity, compromising respiratory efficiency through both mechanical obstruction of the gill structures and reduction in oxygen diffusion capacity. Furthermore, with aeration and water circulation, the interaction between polysaccharides on the epidermal surface and mucoprotein secretions resulted in excessive foam formation and creation of an anoxic environment. Comparative evaluation demonstrated that Groups 9–11 exhibited significantly prolonged survival durations relative to Group 2 (*p* < 0.05), while Group 10 showed particularly remarkable performance, maintaining a superior survival rate throughout the experimental period. Statistical analysis revealed that Group 10 achieved a 72 h survival rate of 50.0%, significantly outperforming all other anti-stress-agent treatment groups (*p* < 0.05). These findings suggest that taurine, when compared with other anti-stress compounds, exhibits enhanced synergistic efficacy with corona dormancy, effectively optimizing both the survival duration and survival rates of *Trachinotus ovatus* during live transport. Comparative analysis revealed that Groups 12–13 demonstrated significantly prolonged survival duration and enhanced survival rates relative to Group 2 (*p* < 0.05). Comparative analysis demonstrated superior efficacy of the 5 mg/L concentration over 10 mg/L, indicating that lower concentrations of thyme oil are more suitable for synergistic applications with corona-induced dormancy technology in aquatic live transportation systems. However, Group 14 exhibited reduced survival parameters, with notable declines in 36 h and 48 h survival rates, attributable to the volatile nature of thyme oil. The progressive volatilization of thyme oil resulted in suboptimal maintenance concentrations, while excessive doses induced profound narcosis, leading to respiratory arrest and subsequent hypoxia-mediated mortality, thereby negating the beneficial effects of corona dormancy. In contrast, Groups 15–17, treated with clove oil, showed improved 36 h survival rates but reduced overall survival duration. This finding demonstrates that the potent anesthetic properties of clove oil render it unsuitable for application in live fish transportation protocols, making it more suitable for commercial anesthesia and euthanasia applications rather than prolonged live transport scenarios.

In summary, the optimal protocol for *Trachinotus ovatus* live transport was established as follows: following a 6 h fasting acclimation period, under an aqueous environment at 20 °C, when specimens were treated for 4 s with parameters of an electric shock voltage of 140 V, an electric shock current of 1 A, an output frequency of 30 Hz, and a duty cycle of 25% in fresh seawater with a volume twice that of the specimens and containing taurine at a concentration of 70 mg/L, the maximum survival time reached 67.7 ± 10.0 h. This represents an 85.99% increase in survival time compared with fish without any treatment, demonstrating the greatest live transportation efficiency.

### 2.2. Influence of Corona Dormancy Combined with Taurine on the Serum Biochemical Indexes of Trachinotus ovatus

#### 2.2.1. Glucose (GLU) and Cortisol (COR) Contents Changes

**Figure 1:**
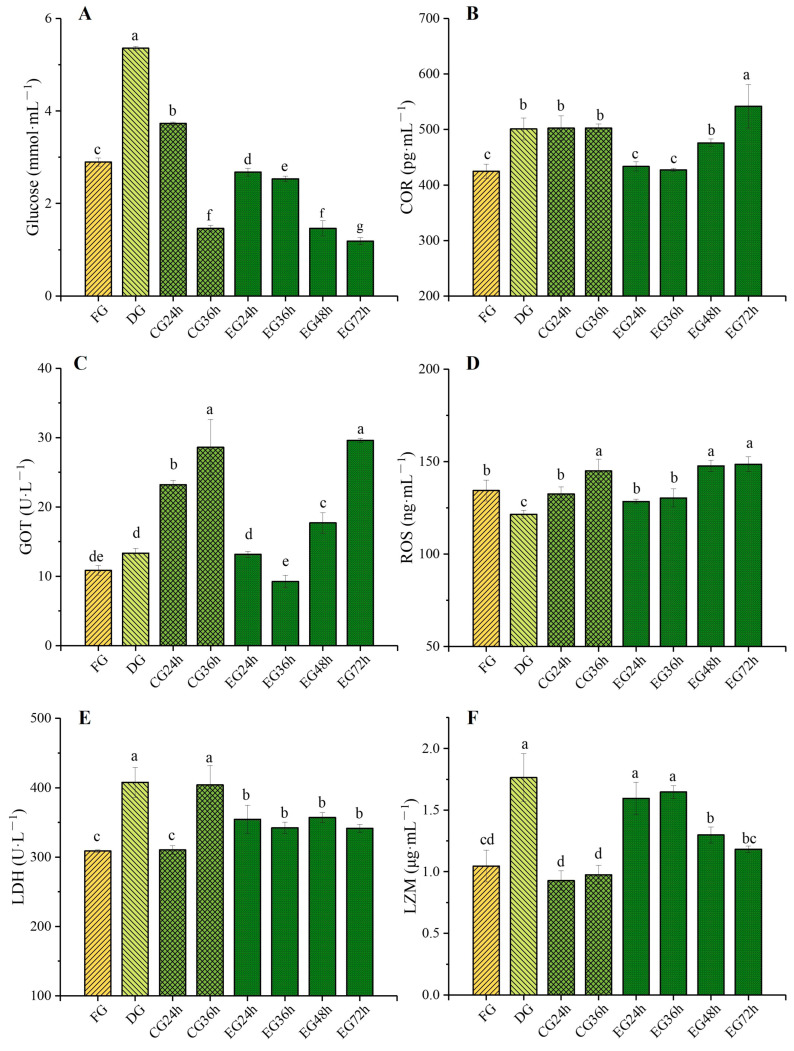
Analysis of biochemical indicators in serum (GLU (**A**), GOR (**B**), GOT (**C**), ROS (**D**), LDH (**E**), LZM (**F**) content changes). Note: Different letters indicate significant differences among groups (*p* < 0.05). (*N* = 6 independent experiments).

As shown in Figure 1, the concentrations of glucose (Figure 1A) and cortisol (Figure 1B) in the blood of fish significantly increased (*p* < 0.05) after corona dormancy treatment compared with the fresh group (FG). Specifically, the dormancy group (DG) showed an increase of 84.8% and 17.9%, respectively, compared with the FG. In the control group (CG), the blood glucose and cortisol concentrations at 24 h were elevated by 28.6% and 18.3%, respectively, compared with the FG. The possible reason was that the transition from the temporary rearing environment to the live preservation environment with less water and gradual cooling intensified the stress level of the fish. However, in the experimental group (EG), the blood glucose concentration at the 24 h and 36 h time points was significantly lower than that of the FG and CG level during the same period (*p* < 0.05), and the cortisol concentration showed no significant difference from that of the FG. The results demonstrate that although electrical stimulation induces a transient stress response, the fish can rapidly restore physiological homeostasis. Furthermore, the corona dormancy state reduces the organisms’ sensitivity to environmental stressors, including low-temperature conditions and reduced water availability, thereby enabling gradual physiological adaptation to new environmental challenges.

#### 2.2.2. Glutamic Oxaloacetic Transaminase (GOT) and Reactive Oxygen Species (ROS) Contents Changes

As depicted in Figure 1C, Serum GOT activity in the dormant group of *Trachinotus ovatus* after electrical stimulation was elevated compared with that of the FG, and meanwhile, the GOT activity in the serum of the CG rose as the survival time prolonged. At 24 h of survival, it was significantly increased by 114% compared with FG (*p* < 0.05), reaching a maximum of 28.61 U/L at 36 h. In contrast, at the two time points of 24 and 36 h of survival, the GOT activity in the serum of the EG was significantly lower than that of the CG group during the same period. However, as the survival time continued to extend, the GOT activity in EG at 48 and 72 h kept increasing, and it reached the level of CG at 36 h at 72 h. Moreover, according to the data, corona dormancy could significantly reduce the content of ROS in fish serum (*p* < 0.05). As the survival time prolonged, the concentration of ROS (Figure 1D) in the serum showed an upward trend. However, the addition of taurine slowed down the accumulation rate of ROS. At 36 h of survival, the ROS concentration of the EG was significantly lower than that of the CG level (*p* < 0.05), being reduced by 11.19%. The results indicated that electrical stimulation induced hepatic alterations in fish, resulting in the release of GOT into the serum. Prolonged live transportation exacerbated oxidative stress due to the accumulation of excretory metabolites and renal damage from low-temperature stress. However, aqueous taurine supplementation effectively mitigated ROS accumulation, enhanced antioxidant capacity, and preserved tissue cellular integrity.

#### 2.2.3. Lactate Dehydrogenase (LDH) and Lysozyme (LZM) Contents Changes

As shown in Figure 1E, corona dormancy could significantly increase the activity of LDH in fish blood. Moreover, during the survival period, the activity of LDH in the EG remained stable and was always significantly higher than that in the fresh group (*p* < 0.05). In the CG, at the 36 h survival time node, the activity of LDH in fish blood was significantly increased compared with that at 24 h (*p* < 0.05), reaching 404.14 U/L, with an increase of 23.19%. At the same time node, it was 18.08% higher than that of the EG. Simultaneously, corona dormancy could significantly enhance the activity of LZM (Figure 1F) in fish blood (*p* < 0.05), reaching 1.76 μg/mL during the dormant period and showing an increase of 67.62% compared with FG. As can be observed from the figure, the LZM activity of the EG generally presented a downward trend during the survival period. Nevertheless, at the 24 h and 36 h survival time nodes, the LZM activity of the EG was still significantly higher than that of the CG, with increases of 72.04% and 70.10%, respectively. The data demonstrated that corona-induced dormancy caused transient hypoxia in fish, while taurine supplementation, acting as an osmoregulator, enhanced gill oxygen exchange efficiency and reduced anaerobic metabolic activity. To mitigate cellular and tissue damage, the piscine immune system responds by maintaining ROS homeostasis. Corona-induced dormancy activates immune responses through physiological stimulation, while taurine, as an immunostimulant, sustains lysozyme activity during live preservation [19].

### 2.3. Influence of Corona Dormancy Combined with Taurine on Oxidative Stress Indexes (Malondialdehyde (MDA), Superoxide Dismutase (SOD), and Glutathione S-Transferase (GST)) in the Brain and Liver of Trachinotus ovatus

**Figure 2:**
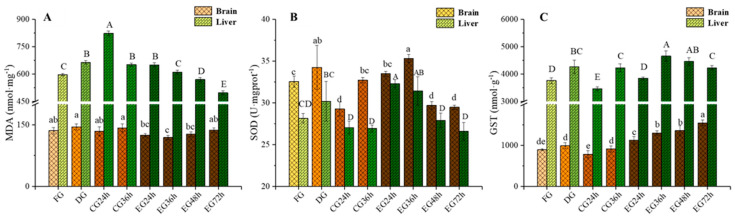
Analysis of biochemical indicators in tissue (MDA (**A**), SOD (**B**), GST (**C**) contents changes). Note: Different letters indicate significant differences among groups (*p* < 0.05). (*N* = 6 independent experiments).

The experimental results (Figure 2A) indicated that after corona dormancy treatment, the content of MDA in the liver was significantly elevated (*p* < 0.05). The DG showed an increase of 11.36% compared with the FG. Moreover, the content of MDA in the liver of the CG reached the maximum value of 823.82 nmol/mg at 24 h of survival. Meanwhile, during the survival period, the content of MDA in the liver tissue of the EG generally exhibited a downward trend and dropped to the lowest level at 72 h. During the survival period, the change in the content of MDA in the fish brain tissue was generally stable and remained lower than the FG level for an extended period. Electrical stimulation may induce hepatic damage in fish, which aligns with the elevated GOT levels observed previously. However, hypothermal and water-restricted conditions exerted a more pronounced oxidative impact on hepatic tissues. Taurine supplementation in the EG significantly mitigated these effects by regulating ROS concentration and enhancing antioxidant enzyme activity.

As shown in Figure 2B, corona dormancy could significantly enhance the activity of superoxide dismutase (SOD) in fish liver and brain tissues (*p* < 0.05). It can be observed that at 24 and 36 h of survival, the SOD activity in both tissues of the EG was significantly higher than that of the CG (*p* < 0.05). Additionally, the SOD activity in the brain and liver tissues of the EG reached its maximum at 36 h and 24 h of survival, respectively, with an increase of 19.42% and 7.91% compared with the CG at the same time period. However, as the survival time prolonged, the SOD activity in each tissue of the EG showed a downward trend. The data demonstrated that taurine supplementation enhanced SOD synthesis in fish. Tissue SOD activity exhibited an inverse correlation with serum ROS levels, indicating SOD-mediated maintenance of ROS generation–scavenging equilibrium.

As can be derived from Figure 2C, corona dormancy could significantly enhance the activity of GST in the liver (*p* < 0.05), with an increase of 13.4% compared with the FG. At the 24 h and 36 h survival time points, the GST activity in the two tissues of the EG was significantly higher than that of the CG at the same time period (*p* < 0.05). Additionally, the GST activity in the liver and brain tissues of the EG showed an upward trend as the survival time extended, and it reached the maximum values of 4658.98 and 1547.72 nmol/g at 36 and 72 h of survival, respectively, with an increase of 23.87% and 73.53% compared with the FG. GST, a pivotal antioxidant enzyme, mediates tissue defense against external stressors. Experimental data demonstrated that GST activity was upregulated in fish under both electrical stimulation and hypothermal/water-restricted conditions to counteract these stressors. Taurine supplementation further amplified GST activity, confirming its synergistic role in enhancing oxidative defense.

### 2.4. Histopathology Analysis

**Figure 3:**
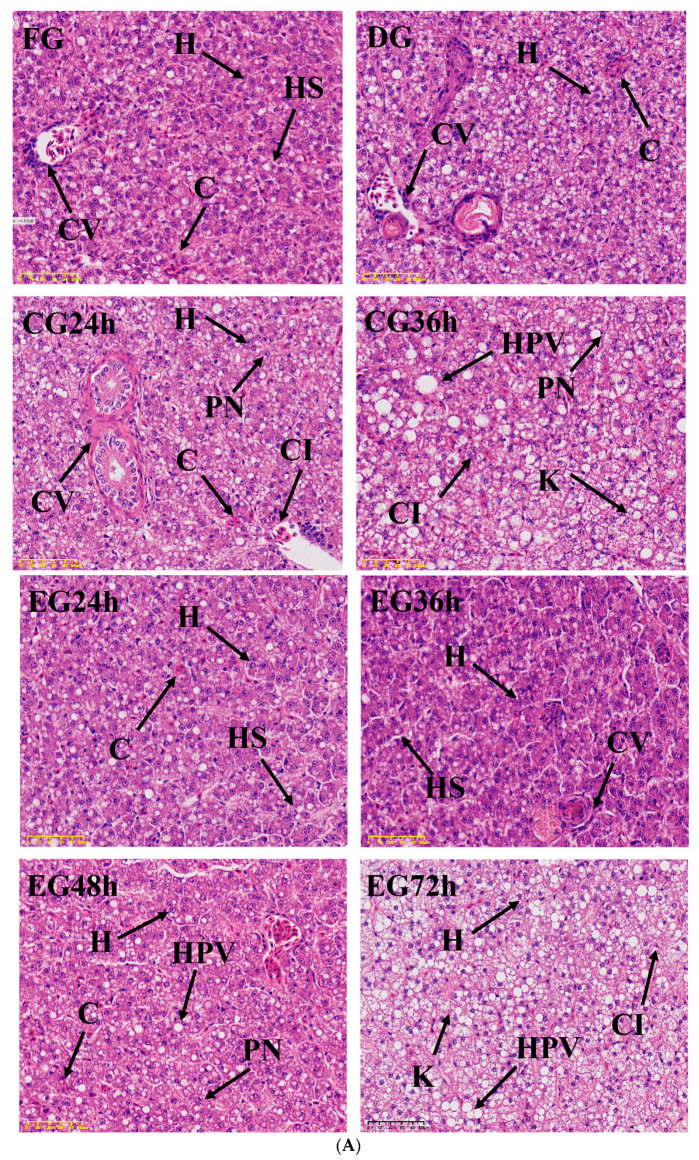

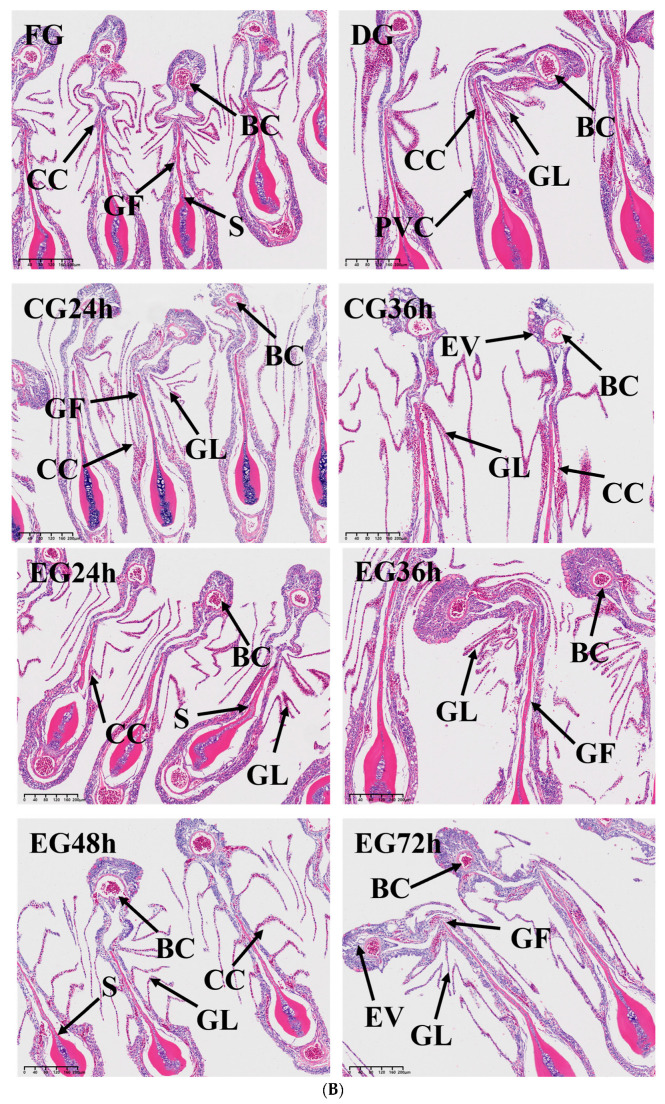
(**A**) The effect of corona dormancy combined with taurine on liver tissue after live transportation. H, hepatocyte; HS, hepatic sinusoid; PN, cellular peripheral nucleus; K, karyolysis; CV, central vein; CI, cellular outline indistinguishable; HPV, hepatocellular vacuolation. (**B**) The effect of corona dormancy combined with taurine on fish gill tissue after live transportation. CC, chlorine cells; EV, cellular vacuolation; PVC, pavement cells; S, blood sinusoid; GL, gill lamellae; GF, gill filaments; BC, blood cell.

It was discovered that in the liver tissue of the FG, the hepatocytes surrounding the central vein were neatly arranged, and the hepatic sinusoids were clearly visible (Figure 3A). The nuclei of hepatocytes within the field were located in the middle of the cells, with clear and intact structures. In the case of the DG, there was partial congestion, and a small quantity of cytoplasmic vacuolities were present. As shown in the CG, as the time of live preservation increased, phenomena such as nucleus displacements, cell vacuolization, and disintegration gradually increased. And in the EG, at the 24–36 h stage of survival, the hepatocytes were closely arranged and the structure was relatively complete. At the 48–72 h stage of survival, the liver tissue structure exhibited variation phenomena. Some nuclei were displaced and dissolved, the cell contours became blurred, and the number of hepatic sinusoids decreased and was difficult to observe.

The synergistic live preservation exerted a remarkably positive influence on the gill tissue structure of *Trachinotus ovatus* (Figure 3B). With the action of hematoxylin and eosin staining solution, the cell nucleus was dyed blue-purple, while the cytoplasm, blood cells, epithelial cells, and others were stained red or light red. As shown in Figure 3, the gill lamellae of the FG were complete and extended to both sides, and the red blood cells were evenly distributed. The physiological state of the gill lamellae of the DG was normal, and there was no obvious difference compared with the FG. In the 24–36 h stage of survival for the CG, it can be seen that the gill lamellae were curled and broken, the chloride-secreting cells proliferated and expanded, the epithelial cells proliferated and swelled, and the number of red blood cells sharply decreased. In the three stages of 24, 36, and 48 h of survival for the EG, there was no obvious difference compared with the FG as a whole. When the EG was kept alive for 72 h, the gill filament epithelium was necrotic, the gill lamellae were seriously damaged, cell vacuolization was aggravated, and the toughness of the gill filaments decreased, resulting in easy breakage after bending.

### 2.5. Impact of Corona Dormancy on the Nrf2-Keap1 Signaling Pathway and the Expression of Genes Related to Antioxidant Stress in the Brain and Liver of Trachinotus ovatus

#### 2.5.1. mRNA Expression Levels of Nrf2 and Keap1 Changes

**Figure 4:**
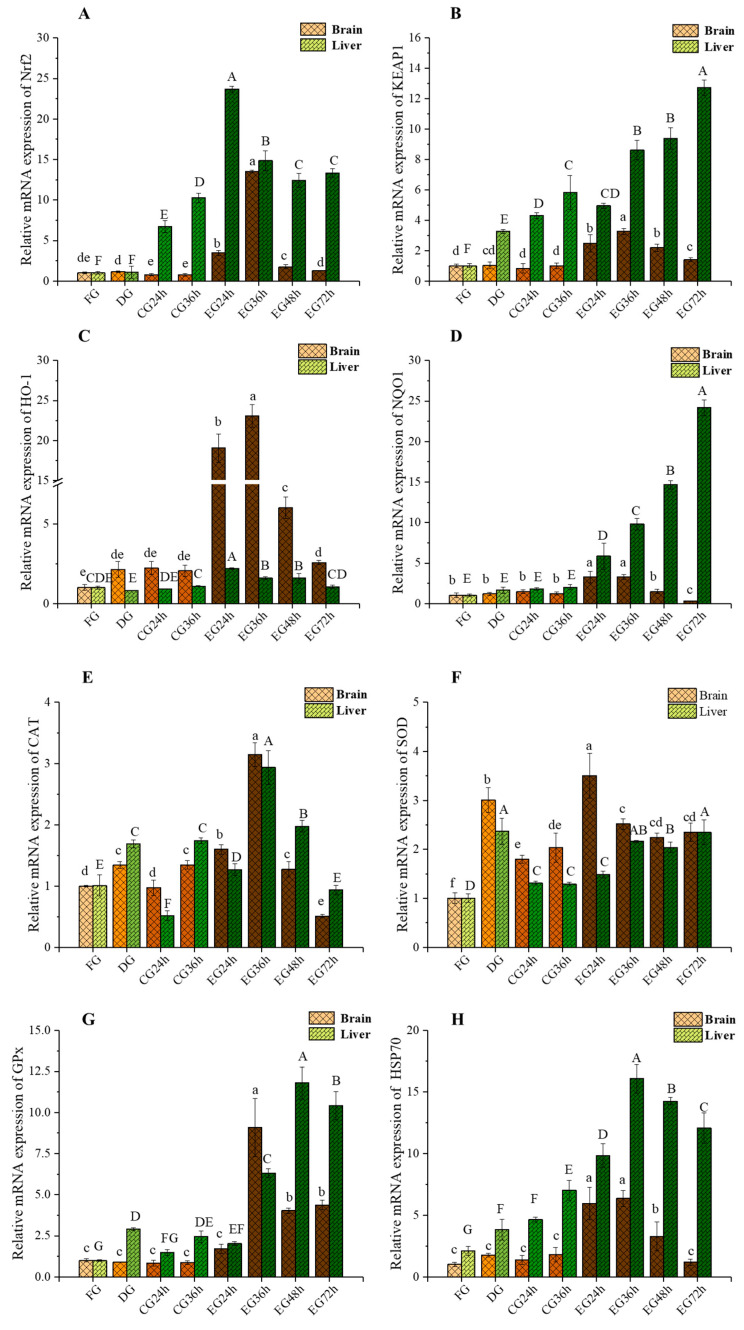
Total RNA extraction, reverse transcription, and real-time PCR (Nrf2 (**A**), KEAP1 (**B**), HO-1 (**C**), NQO1 (**D**), CAT (**E**), SOD (**F**), GPx (**G**), HSP70 (**H**)). Note: Different letters indicate significant differences among groups (*p* < 0.05). (*N* = 6 independent experiments).

As shown in Figure 4, taurine significantly upregulated the mRNA expression levels of Nrf2 (Figure 4A) and Keap1 (Figure 4B) in the fish brain tissue (*p* < 0.05). Throughout the live preservation period, there was a trend of first increasing and then decreasing, and both reached the highest level at 36 h of survival. In contrast, for the DG and CG, compared with the FG, there were no significant changes in gene expression levels at each time point of survival. At the same time, it can be seen from the expression level of gene transcripts in the liver tissue of the CG that low-temperature water could significantly upregulate the mRNA expression levels of Nrf2 and Keap1 in the livers of fish (*p* < 0.05). Compared with the level of the CG, taurine could upregulate the expression levels of the two genes in the liver tissue more significantly (*p* < 0.05). However, the change trend of the Keap1 mRNA transcript level in the liver of the EG fish was opposite.

#### 2.5.2. mRNA Expression Levels of Heme Oxygenase-1 (HO-1) and NAD(P)H: Quinone Oxidoreductase 1 (NQO1) Changes

As shown in Figure 4C, corona dormancy significantly increased the expression level of HO-1 mRNA only in the brain tissue of *Trachinotus ovatus* (*p* < 0.05). Compared with the CG, adding taurine to the water body significantly upregulated the expression levels of the HO-1 and NQO1 (Figure 4D) mRNA transcripts in the brain and liver tissues of fish (*p* < 0.05). For the two genes in the EG brain tissue, during the live preservation period, they generally showed a trend of first increasing and then decreasing, and both reached the highest level at 36 h. Compared with the CG at the same time, the expression levels of the two genes in the EG brain tissue were increased by 10.2-fold and 1.65-fold, respectively. However, the change in the expression levels of these two genes in EG livers showed an opposite trend, reaching the highest level at 24 h and 72 h of survival, respectively.

#### 2.5.3. mRNA Expression Levels of Catalase (CAT) and SOD Changes

As shown in Figure 4E and Figure 4F, respectively, the corona dormancy technology could significantly increase the expression levels of CAT and SOD mRNA in the brain and liver of *Trachinotus ovatus* (*p* < 0.05). At each time point during the survival process, the expression levels of the two genes in the brain and liver of the CG were significantly lower than those of the EG (*p* < 0.05). The expression level of CAT mRNA in the two tissues of the EG was the highest at 36 h of survival, being 68.97% higher and 13.33-fold higher, respectively, than that of the CG in the same period. The mRNA expression levels of SOD in the brain and liver tissues of the EG showed opposite trends during the survival process, reaching the highest levels at 24 h and 72 h of survival, respectively. The experimental results revealed that the changes in SOD mRNA levels in cerebral and hepatic tissues during live preservation were consistent with the previously measured SOD activity. Both electrical stimulation and taurine supplementation significantly upregulated CAT mRNA expression in fish tissues, thereby playing a critical role in cellular protection through enhanced antioxidant enzyme regulation.

#### 2.5.4. mRNA Expression Levels of Glutathione Peroxidase (GPx) and Heat Shock Protein 70 (HSP70) Changes

As depicted in the figure, the corona dormancy technology could notably enhance the transcript expression levels of GPx (Figure 4G) and HSP70 (Figure 4H) mRNA in the liver tissue of *Trachinotus ovatus* (*p* < 0.05). The mRNA expression levels of GPx in the brain and liver tissues of the EG were significantly higher than those of the CG after 36 h of survival (*p* < 0.05), reaching their maximum values at 36 and 48 h of survival, respectively. Simultaneously, at each time point during the survival process, the expression levels of the HSP70 (Figure 4H) mRNA gene in the two tissues of the EG exhibited the same changing trend and were significantly higher than those of the CG. All of them were upregulated to the highest level at 36 h of survival, exceeding by 25.33-fold and 12.94-fold the CG level in the same period, respectively. Hsp70, a reparative protein in tissues, mediates the restoration of cellular functional architecture. Similarly, antioxidant enzymes including GPx and CAT confer organismal protection. The experimental data demonstrated that taurine supplementation upregulated Hsp70 and GPx mRNA expression, thereby enhancing tissue repair capacity, antioxidant defenses, and environmental stress tolerance while mitigating histological damage progression.

## 3. Discussion

### 3.1. Effects of Different Anti-Stress Agents on the Live Transportation of Trachinotus ovatus

Through behavioral observation during the survival process, it was evident that although high-concentration vitamin C could prolong the survival time of fish, in a high-concentration medium, the pH of the seawater was low. The acidic water body led to corrosion and damage to the fish’s epidermal surface, severely impacting the appearance quality. Meanwhile, astragalus polysaccharide is brownish-yellow with a fine powder texture. Its addition resulted in turbidity of the water body, which could cause difficulty in breathing for fish, and it reduced the efficiency of oxygen production in the water. After the polysaccharide combined with the epidermal mucus protein of fish, under the operation of oxygen generators and circulation pumps, the foam in the survival equipment increased, aggravating the occurrence of hypoxia [28]. Furthermore, it was challenging to control the concentration ratios of the essential oils. Given their characteristics of volatility and adhesion, the content of essential oils in the water body gradually decreased as the survival time extended. Moreover, if the added concentration was excessively high, the fish would enter a state of deep dormancy. The cessation of breathing could lead to death due to hypoxia, thereby affecting the survival effect of aquatic products.

### 3.2. Effects of Synergistic Live Preservation on Serum Biochemical Indexes of Trachinotus ovatus

The concentration of GLU in fish blood can be used to measure the level of glucose metabolism in fish. Meanwhile, when fish are under stress, the liver conducts gluconeogenesis in the stress mechanism of the hypothalamic–pituitary–interrenal (HPI) axis. This further increases blood sugar content and accelerates the body’s consumption of blood sugar to counteract stress [29]. Cortisol is an organic compound extracted from the adrenal cortex that possesses various pharmacological effects including anti-inflammation, anti-allergy, and anti-shock. When fish are exposed to external stressors, a significant amount of cortisol is released. Consequently, changes in blood sugar and cortisol can serve as crucial indicators of the stress state of organisms [30]. The results showed that corona dormancy could significantly reduce the metabolic level of fish. At the same time, taurine could promote the stress resistance of golden pomfret by regulating the stress axis and blunting the stress response, thereby effectively reducing the stress caused by electrical stimulation and enabling the fish to quickly adapt to the environment of low water volume and low temperature [31]. For the EG, at the 72 h juncture, blood sugar plummeted to its nadir while cortisol concentration soared to its zenith. The underlying cause was that protracted fasting for live preservation led to the exhaustion of energy. Furthermore, as the duration of live preservation lengthened, the accumulation of ammonia nitrogen deteriorated the aquatic environment, thereby escalating the degree of stress.

GOT is a crucial enzyme in the liver that links the metabolism of sugars, lipids, and proteins. When the liver is injured, a large quantity of GOT is released into the bloodstream, leading to a remarkable increase in its activity. ROS are partially reduced metabolic derivatives of oxygen. When fish are exposed to external environmental stressors or pathogenic infections, excessive ROS generation may occur. Although the cellular antioxidant defense system can mitigate the negative impacts of elevated ROS levels, detrimental effects may still manifest in fish when ROS concentrations exceed the cellular antioxidant capacity [32]. As can be seen from the experimental results, the DG could remarkably reduce the content of reactive oxygen species (ROS) in fish blood (*p* < 0.05), showing a 10.6% decrease compared with the FG. This might be due to the fact that the fish body may activate the antioxidant enzyme system through electrical stimulation, enhancing its ability to resist oxidative stress and thereby reducing the production of ROS. However, in the subsequent live preservation process, the concentration of ROS in serum rose with the prolongation of time. At the 36 h time node, the addition of taurine attenuated the accumulation rate of ROS in fish blood. This was due to the fact that prolonged live preservation led to a gradually deteriorating water environment [33]. Additionally, environmental stressors such as low temperature caused damage to the kidneys of the fish, intensifying the degree of oxidative stress. Taurine could assist in removing reactive oxygen species and other oxidative harmful substances from the organism, enhance the antioxidant capacity of the organism, and protect the fish from oxidative damage [34].

LDH can catalyze the interconversion between pyruvate and lactic acid and participate in anaerobic glycolysis and gluconeogenesis of sugar to provide energy for life activities. Its activity reflects the intensity of anaerobic respiration and the degree of tissue damage [35]. Typically, the activity of LDH increases with the extension of stress time, indirectly indicating that the aerobic respiration of fish is insufficient in providing energy, leading to intensified anaerobic respiration and exacerbating tissue hypoxia. In a hypoxic environment, the activity of LDH is induced to increase, catalyzing the conversion of lactic acid to pyruvate, reducing the accumulation of lactic acid in muscles and the liver, and protecting tissues from hypoxic damage [36]. LZM is a hydrolytic enzyme widely existing in the body fluids, serum, tissues, and organs of aquatic animals that can destroy the structures of bacteria, viruses, parasites, and the like. It is one of the crucial non-specific immune indicators in fish [37]. The above findings suggested that corona dormancy technology may lead to hypoxia in fish. Taurine can facilitate the utilization of energy substances such as glucose by fish bodies. Simultaneously, it can enhance the stability of cell membranes to improve hypoxia tolerance. This achieves the effects of effectively controlling the energy metabolism rate within the fish and reducing the extent of anaerobic respiration reactions to prevent the accumulation of lactic acid. Moreover, taurine is an efficient regulator of immune responses. It can remarkably enhance the activity of LZM in fish serum by modulating the proliferation of lymphocytes, thus improving its non-specific immune parameters [38]. This is beneficial for reducing the oxidative stress in fish induced by environmental stressors such as low temperature and limited water supply, alleviating the decline in their immunity and increasing the live preservation time and survival rate [39].

### 3.3. Effect of Synergistic Live Preservation on Oxidative Stress Indicators in the Brain and Liver of Trachinotus ovatus

MDA is a product of lipid peroxidation in cells and can reflect the degree of oxidative damage to the organism. It is also closely related to the generation of excessive reactive oxygen species [40]. Studies have shown that corona dormancy might cause transient damage to the liver of fish [27]. When the environmental temperature of fish is too low, the antioxidant defense ability of their bodies is damaged, which deepens the degree of lipid peroxidation. Compared with the level before live transportation, the MDA level of the CG was significantly increased. This indicated that low-temperature stress led to the production of a large amount of reactive oxygen species in the fish, causing an increase in the MDA level in their liver tissues. Conversely, the MDA level in EG showed a steady decline. This might be attributed to taurine’s gradual removal of reactive oxygen species in fish directly or indirectly, which slowed down the accumulation rate of ROS in their bodies and thus prevented lipid peroxidation [41].

SOD possesses biological functions such as scavenging reactive oxygen species and mitigating inflammation, and it is ubiquitously present in animals and plants. SOD can eliminate superoxide anions (·O2^−^) through the disproportionation reaction. The ·O2^−^ carries one unpaired electron and one negative charge, and it is highly prone to causing oxidative damage to cells, thereby triggering oxidative stress in the organism [42,43]. Research indicates that changes in SOD activity in tissue cells could precisely reflect the status of free radical clearance and cell damage in fish [44]. When fish are subjected to electrical stimulation or enter a low-water environment without employing dormancy technology, stress-induced ROS continuously accumulate in the body. The addition of taurine in the water helps the organism synthesize SOD, maintaining a balance between ROS production and clearance. This prevents the outbreak of metabolic disorders and oxidative stress, achieving the effect of reducing the degree of damage to the fish [45,46].

GST is an antioxidant enzyme predominantly distributed in the liver. It carries out the dual functions of eliminating peroxides and detoxifying. It can reduce the reactive oxygen species induced by environmental stressors such as low temperature, high salt, and heavy metals, which have toxic effects, thereby minimizing damage to cell structure and function and protecting cells from oxidative damage [47]. During the live preservation process of fish, the following situations were observed. Experimental data indicated that as the live preservation time elapsed, the water environment gradually deteriorated. In addition, the environmental stress on fish caused by low temperature led to the activation of GST in tissue organs to address the oxidative stress pressure induced by the stress, as reported by Pretto et al. (2011) [48].

### 3.4. Effect of Synergistic Live Preservation on the Liver and Gills of Trachinotus ovatus

The liver is a key link in material metabolism and immune protection. It plays a role in resisting or mitigating stress damage when confronted with stress. Its changes can be used as an indicator biomarker for organisms exposed to environmental stressors [49]. The gill is the primary respiratory organ of fish, possessing functions such as oxygen intake, ammonia nitrogen excretion, and osmotic pressure regulation. As an organ with a large surface area in contact with the external environment, it is highly sensitive to environmental changes. Its condition can be regarded as an evaluation indicator for water quality [50]. The results indicated that electric shock could result in local congestion of liver tissue. As the live preservation time extended and the water environment deteriorated, the consumption of energy in the liver tissue might give rise to vacuolization. Additionally, the oxidative stress triggered by low-temperature stress caused liver cells to become increasingly turbid and swollen, with manifestations such as karyolysis and karyomegaly. As a nutrient agent, taurine can replenish energy, and thus, improve the liver condition. Its antioxidant function also serves to delay liver damage [51]. At the same time, although a low temperature weakens the metabolism of fish, a large amount of energy is still needed to maintain osmotic balance during the live preservation process. Relevant studies have shown that temperature stress could stimulate changes in the breathing patterns of fish [52]. Consequently, for the CG, more pronounced hyperplasia occurred at 36 h of live preservation, accompanied by an increase and enlargement in the number of vacuoles as well as the appearance of epidermal shedding. However, in contrast to the CG, for the EG, although the distance between gill filaments gradually increased during 24 to 48 h of live preservation, their condition was robust and intact. The forms of red blood cells and chloride-secreting cells were complete, and there were relatively few vacuoles. The length of gill lamellae showed a gradually increasing tendency. The possible reason was to increase the contact area with water and thereby take in more taurine from the water to maintain osmotic balance. Taurine helped maintain osmotic pressure, reduced the energy consumption of the gill tissue cells, and alleviated tissue damage. Simultaneously, it also proved that corona dormancy slowed down the metabolic rate of the fish and maintained the cleanliness of the water for an extended period.

### 3.5. Effect of Synergistic Live Preservation on the Nrf2-Keap1 Signaling Pathway and the Expression of Antioxidant Stress-Related Genes in Trachinotus ovatus

The Nrf2-Keap1 signaling pathway represents one of the most canonical regulatory mechanisms in the antioxidant systems of aquatic organisms. Under conditions where aerobic cells persistently encounter oxidative damage induced by endogenous metabolic processes and exogenous environmental stressors, cells have evolved an adaptive molecular program activated by reactive molecules to maintain redox homeostasis and minimize macromolecular damage, thereby dynamically enhancing antioxidant capacity [53]. Endogenous Nrf2 serves as a pivotal cytoprotective mechanism within the brain, exerting a central regulatory role in cerebral energy metabolism while simultaneously counteracting oxidative stress and inflammatory responses [54]. The Nrf2 transcription factor exerts a major role in regulating antioxidant enzymes. Keap1, as the upstream central negative regulator of Nrf2, can suppress Nrf2 and its transcriptional level. Heme oxygenase-1 (HO-1) and NAD(P)H: quinone oxidoreductase 1 (NQO1) are two representative downstream genes of Nrf2. As common counteracting factors in tissues against oxidative stress and inflammatory stress, HO-1 can cause partial degradation of heme and generate bilirubin, which is capable of scavenging peroxyl radicals and inhibiting lipid peroxidation. NQO1 can maintain coenzyme Q in cell membranes in an antioxidant form (ubiquinol) [55]. It directly reacts with oxygen radicals and inhibits membrane lipid peroxidation to prevent oxidative stress from causing damage to a variety of biological molecules [17]. Catalase (CAT) is a terminal oxidase widely present in animals, plants, and microorganisms. Glutathione peroxidase (GPx) belongs to a phylogenetically related family of redox enzymes and is distributed across all biological domains [56]. Both of these enzymes are key components of the antioxidant defense system. They are capable of catalyzing the product H_2_O_2_ generated by the superoxide anion produced through SOD disproportionation, transforming it into H_2_O and O_2_, thereby reducing its toxic and adverse effects. Consequently, it is generally acknowledged that CAT, GPx, and SOD jointly construct the first line of defense of the organism’s antioxidant defense system [57]. Shock protein 70 (HSP70) is a ubiquitously existing molecular chaperone. It can restore the functional structure of cells through means such as aiding in the physiological folding of newly synthesized polypeptide chains and correcting the misfolding of polypeptide chains. Additionally, it plays a key role in protecting cells from oxidative stress [58,59]. Numerous studies have indicated that under environmental stress, nutrients can augment the expression of HSP70 [60,61].

Relevant studies have shown that when cells were stimulated and generated oxidative stress, Nrf2 would be separated from Keap1 and enter the nucleus, leading to the accumulation of Nrf2 in the nucleus. Simultaneously, Keap1 in the cells might attempt to reestablish regulatory equilibrium over Nrf2, thereby increasing its expression level [62,63]. Nrf2 could activate the activities of antioxidant enzymes such as SOD, CAT, and GPx, as well as the expression of antioxidant stress-related genes such as heat HSP70, by binding to antioxidant response elements (AREs) and small Maf proteins to prevent oxidative stress [64].

Through experimentation, it was known that fish would undergo oxidative stress when subjected to stressors such as electric stimulation and low temperature. The corona dormancy technique could significantly upregulate the expression levels of antioxidant enzyme genes in fish liver tissues (*p* < 0.05). Meanwhile, taurine added to the water in the experimental group could notably increase the mRNA transcription levels of Nrf2, HO-1, NQO1, SOD, CAT, GPx, and HSP70 in brain and liver tissues. This indicated that fish could regulate the transcription of antioxidant-related genes through the Nrf2-Keap1 signaling pathway by ingesting exogenous taurine from the water, thereby enhancing the antioxidant capacity of brain and liver tissue cells. However, as the duration of live preservation extended, the expression levels of some genes were downregulated. This was because of the continuous consumption of nutrients and the deepening of environmental deterioration.

## 4. Materials and Methods

### 4.1. Materials and Chemicals

All the experimental procedures described in this study were approved by the Animal Care Advisory Committee of Guangdong Ocean University in China. The approval was in accordance with relevant ethical and scientific standards to ensure the proper treatment and welfare of the experimental animals. Vigorous live *Trachinotus ovatus* specimens with intact scales and a body weight of (490 ± 30) g were procured from Xiashan Aquatic Product Market (Zhanjiang, Guangdong Province, China). The salinity of the experimental seawater ranged from 20‰ to 25‰, and the temperature was maintained between 22 °C and 25 °C. Concurrently, a water and air circulation system was employed to continuously filter the seawater and supply oxygen, thereby ensuring that the dissolved oxygen mass concentration in the water was greater than 7 mg/L.

VC (R050488), APS (R096829), and thyme essential oil (Thyme Oil, R129841) were procured from Rhawn Chemical Technology Co., Ltd. (Shanghai, China). Taurine (T6017) and clove essential oil (Clove Oil, C823334) were procured from Shanghai Macklin Biochemical Technology Co., Ltd. (Shanghai, China). GLU (A154-2-1) kit, GOT (C010-2-1) kit, GST (A004-1-1) kit, MDA (A003-4-1) kit, LDH (A020-2-2) kit, total protein (TP, A045-2-2) kit, SOD (A001-3-2) kit, and LZM (A050-1-1) kit were procured from Jiancheng Biological Engineering Institute (Nanjing, Jiangsu Province, China). Ethyl 3-aminobenzoate methanesulfonate (MS-222, E10521) was procured from Sigma Aldrich Chemical Co. (St. Louis, MO, USA). COR (mlsh7908) and ROS (mlsh0829) enzyme-linked immunosorbent assay (ELISA) kit were procured from Shanghai Enzyme Linked Biotechnology (Shanghai, China). FreeZol Reagent kit (R711-01), HiScript III RT SuperMix for qPCR (+gDNA wiper) reagent (R323-01), and CamQ Universal SYBR qPCR Master Mix (Q711-02) reagent were procured from Vazyme Biotech Co., Ltd. (Nanjing, Jiangsu Province, China).

### 4.2. Temporary Rearing Treatment

The experiments were all carried out in January 2024 when the air temperature was 18 °C. A total of 184 *Trachinotus ovatus* individuals with similar body lengths and body weights were randomly divided into 17 groups, with 8 fish in each group. A temporary rearing tank was established based on a ratio of fish mass (in kilograms) to seawater volume (in liters) of 1:20, and all experimental fish were subjected to 6 h of fasting and acclimation in seawater at a temperature of 22 ± 1 °C.

### 4.3. Synergistic Live Transportation

The experiment referred to the dosage ranges of anti-stress agents for fish species such as *Trachinotus ovatus*, *Cyprinus carpio*, *Pelteobagrus fulvidraco*, and *Ictalurus punctatus* in previous studies (Fan et al. (2023), Li. (2016), Du. (2022), Qi. (2023), Li et al. (2023)) [65,66,67,68,69]. Preliminary experiments were conducted to determine the concentration. In the experimental group, additives were weighed according to the volume of seawater and were fully mixed. Fresh seawater containing different mass concentrations of VC (10, 25, and 40 mg/L), APS (25, 50, and 75 mg/L), taurine (20, 70, and 120 mg/L), thyme oil (5, 10, and 15 mg/L), and clove oil (5, 10, and 10 mg/L) was prepared.

The static simulation experiment method for in vivo preservation in water was based on previously published research [14]. The specific implementation was as follows. The water temperature of the temporary rearing system was cooled by gradient at a rate of 3 °C·h^−1^ to reach 20 °C. Corona dormancy treatment was applied to the fish body with an output frequency of 30 Hz, a duty cycle of 25%, a voltage of 140 V, and a duration of 4 s. The cessation of gill movement in fish was observed, indicating that the fish had entered a dormant state. The dormant fish were placed in seawater with a fish-to-water ratio of 1:2. Subsequently, the temperature was reduced to 15 °C at a rate of 2 °C·h^−1^ in a constant temperature chamber to conduct the experiment.

### 4.4. Survival Rate and Survival Time of Trachinotus ovatus Under Different Anti-Stress Agents

The experimental design involved dividing *Trachinotus ovatus* into 17 groups, with each group containing 8 specimens, as systematically outlined in Table 1. Following acclimatization under the conditions delineated in Section 2.2, the experimental fish were directly transferred to natural seawater for live preservation, constituting the control group (CG, 1). The experimental fish were subjected to electrically induced dormancy and subsequently transferred to natural seawater for live preservation, establishing the dormancy group (DG, 2). The remaining fifteen experimental groups (Groups 3–17) were established by subjecting the *Trachinotus ovatus* specimens to electrically induced narcosis followed by immediate immersion in seawater containing varying concentrations of VC, APS, taurine, thyme oil, and clove oil, prepared according to the protocols detailed in Section 2.3. In the simulated live transport experiment, a standardized fish-to-water ratio of 1:2 was maintained across all experimental groups to ensure consistent physiological conditions. Throughout the experimental period, survival duration was systematically recorded, with survival rates quantified at predetermined intervals (24 h, 36 h, 48 h, and 72 h). Mortality was determined through comprehensive assessment of three physiological indicators: absence of tail reflex response to tactile stimulation, corneal opacity characterized by whitening and loss of light reflection, and cessation of cardiac activity.

### 4.5. Index Detection

Based on empirical optimization of survival parameters, systematic sampling was conducted at predetermined temporal intervals for comprehensive analysis of relevant physiological and biochemical indicators. The experimental design involved partitioning *Trachinotus ovatus* into 8 distinct groups, each comprising 6 specimens. Following acclimatization under the conditions specified in Section 2.2, the fresh group (FG) was established with specimens subjected to a 6 h acclimation period exclusively. Concurrently, the dormancy group (DG) was designated, comprising specimens that were immediately sampled following electrically induced dormancy, ensuring the preservation of the dormant physiological state. Groups 3 and 4 were maintained in natural seawater at 15 ± 0.5 °C for simulated live transport, designated as the 24 h control group (CG 24 h) and 36 h control group (CG 36 h) based on their respective survival durations. Groups 5 through 8 were subjected to electrically induced dormancy followed by preservation in formulated seawater (70 mg/L taurine concentration) at 15 ± 0.5 °C, systematically categorized as the 24 h experimental group (EG 24 h), 36 h experimental group (EG 36 h), 48 h experimental group (EG 48 h), and 72 h experimental group (EG 72 h) according to their predetermined observation intervals.

#### 4.5.1. Blood Sampling and Determination of Serum Biochemical Indexes

At each sampling time point, five *Trachinotus ovatus* were randomly captured. Subsequently, they were anesthetized using 0.5 g/L MS-222. A disposable medical syringe was employed to collect caudal vein blood and rapidly inject it into a blood collection tube without anticoagulant. Only a single operation was conducted on each fish. During blood collection, repeated punctures should be avoided to prevent hemolysis. After standing for 120 min, the whole blood was centrifuged for 20 min at 4 °C and 3500 revolutions per minute. The plasma was meticulously separated and subsequently stored at 4 °C to ensure preservation of its biochemical integrity for subsequent analytical procedures. The content of GLU, COR, ROS, and LZM in serum and the activity of GOT and LDH were determined according to the instructions of the kit.

#### 4.5.2. Liver and Brain Tissue Sampling and Determination of Oxidative Stress Indicators

Liver and brain tissues were obtained on ice. The blood stains were washed off with precooled physiological saline. After being quick-frozen in liquid nitrogen, they were stored in a −80 °C refrigerator for further testing. The content of MDA and GST in liver and brain tissues and the activity of SOD were determined according to the instructions of the kit.

#### 4.5.3. Histopathology

The liver and gill tissues of the experimental fish that had been fixed were processed into small pieces with an area not exceeding 2 cm^2^. Then, they were dehydrated with gradient ethanol to render the tissues transparent. Immediately, they were embedded in molten paraffin. After hardening, thin slices (4 μm) were cut with a microtome, flattened in hot water, and transferred to a glass slide for drying treatment. The nuclei, cytoplasm, and extracellular matrix of tissues were stained with hematoxylin and eosin dyes. The morphological characteristics of liver and gill tissues were observed using a microscope (CKX41, Olympus, Shinjuku, Japan) at magnification of 400×.

#### 4.5.4. Real-Time Quantitative PCR (qRT-PCR)

β-Actin was selected as the internal reference gene. The primer sources of all tested genes, namely nuclear factor erythroid 2-related factor 2 (Nrf2), Kelch-like ECH-associated protein-1 (Keap-1), heme oxygenase-1 (HO-1), NAD(P)H: quinone oxidoreductase 1 (NQO1), catalase (CAT), glutathione peroxidase (GSH-Px), superoxide dismutase (SOD), and heat shock protein 70 (HSP70), are presented in Table 2. Total RNA was extracted from the brain and liver by means of the FreeZol Reagent kit. Subsequently, the RNA was reverse transcribed into cDNA utilizing the HiScript III RT SuperMix for Qpcr (+Gdna wiper) kit. Finally, the mRNA expression levels were determined by employing the ChamQ Universal SYBR Qpcr Master Mix. Each sample was subjected to quadruplication to eliminate the influence of incidental factors, and three results were randomly selected for analysis. The relative expression level of the target gene was calculated by the 2^−∆∆CT^ method.

### 4.6. Statistical Analysis

All measured data were the average of the results, and the data were presented as mean ± standard deviation. Statistical analyses between samples were performed using one-way analysis of variance (ANOVA) test via SPSS software (Version 26.0, IBM Corporation, Armonk, NY, USA). Combined with Duncan’s analysis for significance of differences and multiple comparisons, *p* < 0.05 was used to indicate significant differences between groups.

## 5. Conclusions

**Figure 5:**
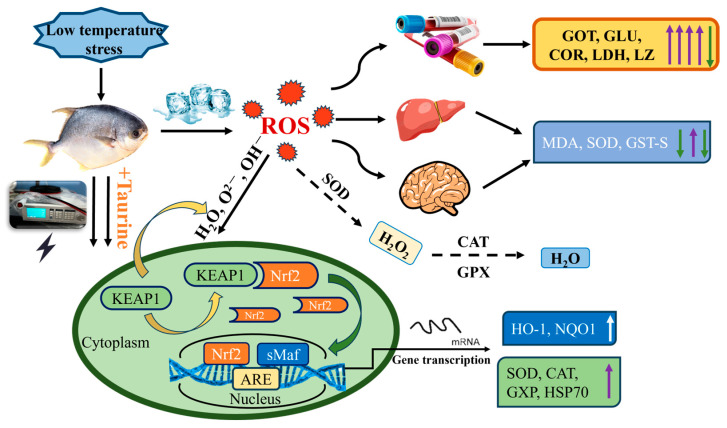
Effects of corona dormancy combined with taurine on the Nrf2-Keap1 pathway.

The low-temperature environment of the water body poses a significant threat to the safety and life of aquatic animals during live transportation. Prolonged exposure to low-temperature water can lead to oxidative damage to the tissues and organs of fish. Additionally, it can affect biochemical parameters, tissue cells, and the expression of related genes. As shown in Figure 5, the results of this study revealed that solely utilizing low temperature for live transportation with limited water volume would significantly accelerate the occurrence of oxidative stress within fish. Conversely, subjecting fish to corona dormancy using pulsed direct current with an output frequency of 30 Hz, duty cycle of 25%, voltage of 140 V, and duration of 4 s, and then placing them in a taurine protective solution with a concentration of 70 mg/L for live transportation, could effectively enhance the activities of anti-stress, defense, and immune enzymes. Additionally, it could regulate the gene expression levels of related antioxidant enzymes via the Nrf2-Keap1 pathway to attenuate the stress response of fish under low-temperature stress and reduce tissue damage. During the live preservation period, fish could maintain a relatively low metabolic level, thereby enhancing efficiency and quality during the live transportation process.

## Figures and Tables

**Table 1:** The influence of anti-stress agents on the survival time and survival rate of *Trachinotus ovatus* in corona dormancy.

Group		Concentration	pH	Keep Alive (h)	24 h Survival Rate	36 h Survival Rate	48 h Survival Rate	72 h Survival Rate
1	CG	0	7.3	36.4 ± 3.5 ^f^	87.50%	37.50%	0%	0%
2	DG	0 mg/L	7.3	47.3 ± 2.4 ^de^	100%	75%	25.00%	0%
3	VC	10 mg/L	6.9	55.7 ± 6.7 ^bc^	100%	100%	62.50%	0%
4		25 mg/L	6.5	58.3 ± 8.1 ^bc^	100%	100%	62.50%	0%
5		40 mg/L	6.2	60.1 ± 5.5 ^b^	100%	100%	62.50%	0%
6	ASP	25 mg/L	7.3	56.5 ± 8.4 ^bc^	100%	100%	50%	0%
7		50 mg/L	7.3	60.3 ± 7.1 ^ab^	100%	100%	62.50%	0%
8		75 mg/L	7.1	43.4 ± 3.7 ^ef^	100%	75%	0%	0%
9	Taurine	20 mg/L	7.3	61.4 ± 8.9 ^ab^	100%	100%	50%	0%
10		70 mg/L	7.2	67.7 ± 10.0 ^a^	100%	100%	100%	50%
11		120 mg/L	7.2	56.2 ± 9.7 ^bc^	100%	100%	62.5%	0%
12	Thyme oil	5 mg/L	7.3	56.4 ± 6.2 ^bc^	100%	100%	50%	0%
13		10 mg/L	7.3	52.3 ± 2.5 ^cd^	100%	100%	62.50%	0%
14		15 mg/L	7.4	44.4 ± 6.4 ^e^	100%	50%	37.50%	0%
15	Clove oil	5 mg/L	7.3	44.3 ± 2.5 ^e^	100%	87.50%	0%	0%
16		10 mg/L	7.3	45.7 ± 2.7 ^de^	100%	87.50%	25%	0%
17		15 mg/L	7.2	44.5 ± 2.0 ^e^	100%	87.50%	0%	0%

Note: Different superscript letters on the parameters in the same column indicate significant differences (*p* < 0.05), while the same letters represent no significant differences. (*N =* 8 independent experiments).

**Table 2:** qPCR primers and source.

Primer	Primer Sequence (5′-3′)	Source
CAT-F	GGATGGACAGCCTTCAAGTTCTCG	Liu et al. (2021) [70]
CAT-R	TGGACCGTTACAACAGTGCAGATG	
SOD-F	CCTCATCCCCCTGCTTGGTA	Liu et al. (2021) [70]
SOD-R	CCAGGGAGGGATGAGAGGTG	
GSH-PX-F	GCTGAGAGGCTGGTGCAAGTG	Liu et al. (2021) [70]
GSH-PX-R	TTCAAGCGTTACAGCAGGAGGTTC	
HSP70-F	TTGAGGAGGCTGCGCACAGCTTGTG	Tan et al. (2017) [71]
HSP70-R	ACGTCCAGCAGCAGCAGGTCCT	
Nrf2-F	TTGCCTGGACACAACTGCTGTTAC	Liu et al. (2021) [72]
Nrf2-R	TCTGTGACGGTGGCAGTGGAC	
HO-1-F	AGAAGATTCAGACAGCAGCAGAACAG	Xie et al. (2020) [73]
HO-1-R	TCATACAGCGAGCACAGGAGGAG	
Keap-1-F	CAGATAGACAGCGTGGTGAAGGC	Liu et al. (2021) [72]
Keap-1-R	GACAGTGAGACAGGTTGAAGAACTCC	
NQO1-F	TGGTCCAGGTGTCACGTCTTCC	Xie et al. (2020) [73]
NQO1-R	GACTTGGCGTGTAGTGCTTGG	
β-Actin-F	TACGAGCTGCCTGACGGACA	Xie et al. (2020) [73]
β-Actin-R	GGCTGTGATCTCCTTCTGCA	

## Data Availability

Data will be made available on request.
